# A New Method for Ivermectin Detection and Quantification through HPLC in Organic Matter (Feed, Soil, and Water)

**DOI:** 10.1155/2023/6924263

**Published:** 2023-03-01

**Authors:** Alicia Maria Carrillo Heredero, Giulia Segato, Simonetta Menotta, Elena Faggionato, Alice Vismarra, Marco Genchi, Simone Bertini

**Affiliations:** ^1^Department of Veterinary Sciences, University of Parma, Strada del Taglio 10, Parma 43126, Italy; ^2^Food and Feed Chemical Department, Experimental Zooprophylactic Institute of Lombardy and Emilia-Romagna, Via Antonio Bianchi, 7/9, Brescia 25124, BS, Italy

## Abstract

Ivermectin is a macrocyclic lactone widely used in veterinary medicine for its broad-spectrum antiparasitic properties. It has been proven to be effective and safe. The purpose of this study was to develop a high-performance liquid chromatography method with a diode array detector for ivermectin screening in feed and water for animal consumption. Furthermore, the objective was to quantify ivermectin levels that were higher than 0.5 mg/kg in solid matrixes and 0.1 mg/kg in water. Doramectin was used as process standard. Samples were extracted using solid phase extraction with silica and C-18 columns. The method involved the use of high-performance liquid chromatography (HPLC) with a diode array detector (DAD). The results were interpreted using a calibration curve built with ivermectin standards at multiple concentrations (0.5, 1, 2, 5, and 12.5 mg/kg). Statistical evaluation of data was done using ANOVA. The data analysis showed that the linear regression was highly significant (*P* < 0.001), the intercept values were not significantly different from zero, and the correlation coefficient values (>0.999) indicated excellent linearity. Further tests demonstrated that this method is also useful when studying soil matrixes. The soil was dried and analyzed in the same way as feed; the same recoveries were realized on the spiked samples. The method is easy, inexpensive, precise, and repeatable; it requires very small amounts of sample.

## 1. Introduction

Ivermectin is a macrocyclic lactone widely used in veterinary medicine for its broad-spectrum antiparasitic properties. Ivermectin is naturally produced by the yeast *Streptomyces avermitilis*, and it consists of a mixture of two compounds: 22,23-dihydroavermectin *β*1a (C_48_H_74_O_14_, B_1a_) and 22,23-dihydroavermectin *β*1b (C_47_H_72_O_14_, B_1b_). Ivermectin B_1a_ makes at least 80% of the mixture, while no more than 20% is made of ivermectin B_1b_. It is active against both arthropods and nematodes [1, 2], causing their paralysis and subsequent death by blocking chloride channels [3]. Like all avermectins, ivermectin is highly liposoluble [4].

Ivermectin is authorized for oral, topical, and parenteral (subcutaneous) administration [1, 2, 5]. The European Medicine Authority has authorized ivermectin for multiple species: cattle, pigs, sheep, goats, horses, reindeers, mice, rats, dogs, cats, primates, and humans. Ivermectin is marketed either alone or in combination with other antiparasitic compounds [6, 7]. In farm animals, doses can vary according to the route of administration and range from 200 *µ*g per kg of body weight in cattle, sheep, and goats to 300 *µ*g per kg of body weight in pigs [6, 8].

It is estimated that between 80 and 98% of the drug is excreted in feces, without being metabolized, thus reaching the environment intact.

Maximum residual limits (MRLs) have been established for all mammalian food-producing species, except animals producing milk for human consumption. The marker residue is 22,23-dihydroavermectin B1a. The MRLs are restricted in the targeted tissues in all species as follows: 30 *µ*g/kg in the kidney and 100 *µ*g/kg in the fat and liver [8, 9]. The Italian National Reference Center for the surveillance and control of feed for animals (CReAA—Centro di Referenza Nazionale per la Sorveglianza e il Controllo degli Alimenti per gli Animali) has established a detection limit of less than 1 mg/kg in feed.

Although ivermectin has a wide safety margin in mammals [1, 2], it has been shown to be toxic in numerous invertebrate species. Mesa et al. reported possible ecological consequences in aquatic ecosystems [10]. Johnson-Arbor has raised environmental concerns about ivermectin residues [11]. Therefore, an accurate system to monitor ivermectin levels in organic substrates would therefore be of crucial importance.

Different methods for ivermectin detection have been reported by several authors. Åsbakk et al. used high-performance liquid chromatography (HPLC) with fluorescence detection to detect ivermectin in feces from reindeers [12]. Iglesias et al. used the same method for soil and cattle feces [13]. Liquid chromatography-tandem mass spectrometry (LC-MS-MS) has also been shown in several studies to be useful for multiple matrixes such as plasma, milk, liver, and whole blood [14–20]. Liquid chromatography-electrospray ionization-mass spectrometry/mass spectrometry (LC-ESI-MS/MS) has also been used to detect macrocyclic lactones in fish muscle [14].

This study aims to develop a HPLC method with a diode array detector (DAD) for screening for ivermectin in feed and water for animal consumption. Furthermore, we wanted to quantify ivermectin levels higher than 0.5 mg/kg in solid matrixes and 0.1 mg/kg in water. We used doramectin as process standard [13].

## 2. Materials and Methods

### 2.1. Instruments

Instruments employed for the experiment consisted of ultrasound water bath (SONICA Sweep System Mod. 5300EP, SOLTEC, Milan, Italy), homogenizer type Grindomix (RETSH Grindomix Mod.GM200, Verder scientific, Haan, Germany), flask shaker (Janke and Kunkel Mod. HS501 digital, IKA Labortechnik, Staufen, Germany), centrifuge (Eppendorf Mod, 5804 R, Merck KGaA, Darmstadt, Germany), vacuum pump (KNF Neuberger, Mod. Laboport N820 FT.18, Milan, Italy), water bath at 50°C ± 5°C with nitrogen current device (Buchi Mod. B-461 water bath, GEMINI LAB sustainable equipment, Apeldoorn, the Netherlands), vortex (Ika MS2 Munishaker, IKA Labortechnik, Staufen, Germany), and scales (Mettler Toledo Mod. XPE12025, Columbus, OH, USA; Sartorius Mod.ME235P, Sartorius, Göttingen, Germany). Of the two scales used, one had 10 mg sensitivity and one had 0.01 mg sensitivity.

The system for high-performance liquid chromatography included a degasser, pump, autosampler, diode array detector, and software Agilent ChemStation Rev. A.10.02 (1757). It was equipped with a chromatographic precolumn (Supelguard for inverse phase, C-18, 2 cm long, internal diameter: 4.6 mm, and particle mean diameter: 5 *µ*m, Supelco, Bellefonte, PA, USA) and a chromatographic column (Supelcosil for inverse phase, C-18, 25 cm long, internal diameter: 4.6 mm, and particle mean diameter: 5 *µ*m, Supelco, Bellefonte, PA, USA).

Smaller equipment employed consisted of plastic adapters for the reservoirs, 50 mL polypropylene tubes with pressure caps (EuroClone S.p.A. Milan, Italy), 50 mL plastic reservoirs, a manifold system suited for solid phase extraction (SPE) (Supelco, Bellefonte, PA, USA), pipettes (10–1000 *µ*L) (Gilson Middleton, WI, USA), 500 mg/6 mL SPE C-18 ENVI columns (Supelco code: 57064, Supelco, Bellefonte, PA, USA), and 500 mg/6 mL SPE silica LC-Si columns (Supelco code: 505374, Supelco, Bellefonte, PA, USA). Disposable materials used were 0.45 *μ*m filters (Incofar, Modena, Italy), 6 mL reservoirs with 13 mm internal diameter (Strata, Phenomenex, Torrance, CA, USA), frits (Phenomenex, Torrance, CA, USA), 10 mL plastic syringes (Rays SpA, Osimo, AN, Italy), and 250 mL plastic centrifuge bottles with screw caps (Nalgene, Rochester, NY, USA).

Glassware used consisted of 10 mL v-bottom graded test tubes, 10 mL u-bottom test tubes, 10 mL–20 mL amber test tubes with screw caps, class A or B glass pipettes, Pasteur glass pipettes, graded cylinders, class A flasks, and amber vials.

### 2.2. Chemicals and Reagents

All reagents had known analytical purity. The water used was deionized and ultrafiltrated water.

Methanol for analysis from Carlo Erba Reagents (DASIT Group S.p.A., Milan, Italy) was used for the extraction, while Aluminium oxide 90 (neutral alumina) from Merck (Merck KGaA, Darmstadt, Germany) was used for the preliminary chromatography phase. The following solvents were used for SPE column activation and elution as well as components of the HPLC mobile phase: methanol for HPLC from Carlo Erba Reagents (DASIT Group S.p.A., Milan, Italy), acetonitrile for HPLC from Carlo Erba Reagents (DASIT Group S.p.A., Milan, Italy), and dichloromethane for analysis from Carlo Erba Reagents (DASIT Group S.p.A., Milan, Italy). Ivermectin with declared purity from Dr Ehrenstorfer (LGC Labor GmbH Augsburg, Germany) and doramectin with declared purity from Sigma-Aldrich (Merck KGaA, Darmstadt, Germany) were used, respectively, for the fortified specimen and as an internal standard.

For each new experiment, a mixture of solvents for dilution made of acetonitrile (56%), methanol (37%), and water (7%) (v/v) and the activated neutral alumina (100 g of neutral alumina moisturized with 6 mL of deionized water) were prepared before use.

All solvents and solutions used for the HPLC system were filtered on a 0.2 *μ*m filter.

### 2.3. Preparation of Standard and Quality Control Samples

All standards and controls were prepared just before use. A stock solution of ivermectin and one of doramectin was prepared in a 10 mL flask, considering the declared purity, at a concentration of 1000 *µ*g/mL. The solution was made with methanol for HPLC and stored in the freezer in an amber tube for no more than 12 months [21].

An intermediate solution was made for each compound at 10 *µ*g/mL, starting from the stock solution. In a 10 mL flask, the stock solution was added to the solvent mixture (acetonitrile (56%), methanol (37%), and water (7%)) at a concentration of 1 : 100.

From the intermediate solutions, ivermectin was diluted with the solvent mixture to obtain five working solutions at 0.1, 0.2, 0.4, 1, and 2.5 *µ*g/mL for calibration points. Another working solution (0.05 *µ*g/mL) was made by adding 50 *µ*L of the intermediate solution of ivermectin and doramectin to 9.95 mL of the solvent mixture.

For each analytical session, a blank control and a fortified sample at 0.1 mg/L were set up for water samples. In a 10 mL flask partially filled with deionized water, 100 *µ*L of intermediate solution at 10 *µ*g/mL was added, made up to volume, and diluted in a ratio of 1 : 2 with a dilution mix.

For each analysis session, a blank control and a fortified control were set up for solid samples (feed or soil). Negative control (blank) consisted of 10 g of an ivermectin-free sample (feed or soil). The fortified sample (0.5 mg/kg) was made of 10 g of an ivermectin-free sample (feed or soil) to which 500 *µ*L of the intermediate solution at 10 *µ*g/mL was added and left for at least 10 minutes.

All samples (controls and tests) were subjected to the same extraction, purification, and analysis procedure.

### 2.4. Experimental Design

The solid samples were extracted with methanol and purified using a solid phase extraction (SPE) technique. The purified extracts were analyzed using HPLC after dilution.

#### 2.4.1. Extraction and Purification of the Sample

The water samples were diluted in a ratio of 1 : 2 with the diluting solution, and 500 *µ*L of the intermediate solution of the internal standard (doramectin) was added at 10 *µ*g/mL; 1.5 mL was transferred in an amber vial.

Soil samples were dried in a heater at 45°C prior to processing. At least 2/3 of the total solid samples (feed or soil) were shredded to obtain a homogeneous powder. Using a scale with a sensitivity of 0.01 g, 10 g of ground samples were transferred to a conical flask. 50 mL of methanol and 500 *µ*L of the intermediate solution of the internal standard (doramectin) at 10 *µ*g/mL were added to the sample, and the flask was capped. The flasks were placed in a water bath with ultrasound for 20 minutes and then shaken on the shaker for one hour at 700 oscillations/minute. 40 mL of supernatant was placed in plastic test tubes with a pressure cap and centrifuged at 3000 rpm for 5 minutes. A frit was put inside each 6 mL reservoir, then 5 g of activated neutral alumina were added, and another frit was put on top. The 50 mL reservoirs were then fixed on top with the appropriate adapters. 15 mL of centrifugate supernatant was added to the 50 mL reservoir. The first 5 mL was discarded, and the remaining volume was collected in glass tubes.

For activation, the SPE silica columns were inserted into the manifold, one for each sample plus the blank and fortified controls. We percolated 5 mL of acetonitrile for HPLC by gravity in columns and then dried them for 1 minute using vacuum. 5 mL of dichloromethane for HPLC was leached and dried for 1 minute after applying vacuum.

For conditioning, the SPE C-18 columns were inserted into a second manifold, one for each sample plus the blank and fortified controls. 5 mL of dichloromethane was percolated by applying vacuum and dried for 1 minute; then 5 mL of a water/acetonitrile mixture (50 : 50 v/v) was percolated, and the column was kept wet.

A 10 mL syringe was applied to the SPE C-18 columns using the appropriate adapter. Simultaneously, 5 mL of the water/acetonitrile mixture (50 : 50 v/v) and 2 mL of each sample were put in each syringe using a calibrated class A pipette. The fluid was allowed to percolate, keeping the column wet. Columns were then washed with 1 mL of the water/acetonitrile mixture (50 : 50 v/v) that was left to percolate by gravity and then dried with vacuum for 15 seconds. The walls of the columns were washed by adding 2 mL of the water/acetonitrile mixture (50 : 50 v/v) and by letting it flow with the use of the vacuum pump. Finally, the column was dried for 1 min, maintaining the vacuum.

The SPE C-18 columns were inserted into the conditioned SPE silica columns with the appropriate adapter. 5 mL of the dichloromethane/acetonitrile mixture (9 : 1 v/v) was added to the SPE C-18 columns and left to percolate, maintaining the vacuum for 1 minute; another 5 mL of the mixture was subjected to the same procedure. The SPE C-18 columns were discarded, and ivermectin was eluted from the silica SPE column with 5 mL of acetonitrile for HPLC by gravity, without letting it go dry. A further 2 mL of acetonitrile for HPLC was added and eluted by applying vacuum. The eluate was collected in a conical tube and dried in a nitrogen stream at 50°C ± 5°C. The eluate was then diluted with 2 mL of the solvent mixture and vortexed for 1 minute, and the samples were filtered on 0.45 pm filters. Approximately 1.5 mL was placed in amber vials.

#### 2.4.2. Analysis by High-Performance Liquid Chromatography

Reverse-phase liquid chromatography was carried out. The mobile phase consisted of a solution containing acetonitrile (56%), methanol (37%), and water (7%) in an isocratic process. The stationary phase consisted of a C-18 precolumn and a C-18 column, respectively, a Supelguard reverse-phase chromatographic column C-18 (2 cm long, 4.6 mm internal diameter, and 5 *µ*m average particle diameter) and a C-18 Supelcosil reverse-phase chromatographic column (25 cm long, 4.6 mm internal diameter, and 5 *µ*m average particle diameter).

All HPLC analyses were conducted at room temperature (20°C). Before injecting the samples, the system was equilibrated for 30 minutes under the conditions described above. The flow was set at 1.2 mL/min, and the wavelength at 245 nm. A volume of 100 *µ*L was injected for each sample run.

**Figure 1:**
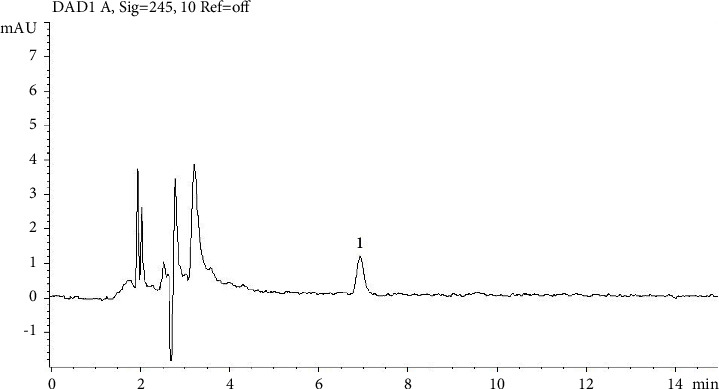
Chromatogram of a blank sample. At ivermectin retention times, no peak was registered, while doramectin's peak is indicated with number 1 (RT 6.929).

**Figure 2:**
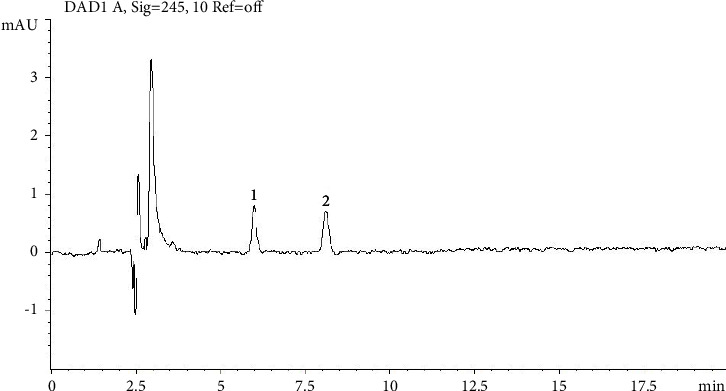
Chromatogram of the spiked sample. The numbers indicate the peaks of interest at respective retention time (RT). Number 1 indicates the peak of doramectin (RT 5.990), while number 2 is ivermectin (RT 8.100).

The ivermectin standard (0.05 *µ*g/mL), the internal standard (0.05 *µ*g/mL), the blank (Figure 1), and the fortified controls (Figure 2) were analyzed first. Subsequently, all samples were run and the standard injections were repeated every three samples.

The analysis was continuously monitored by Accredia, the Italian accreditation body, which certifies the quality of laboratory practices and conformity of the results obtained.

## 3. Results and Discussion

**Figure 3:**
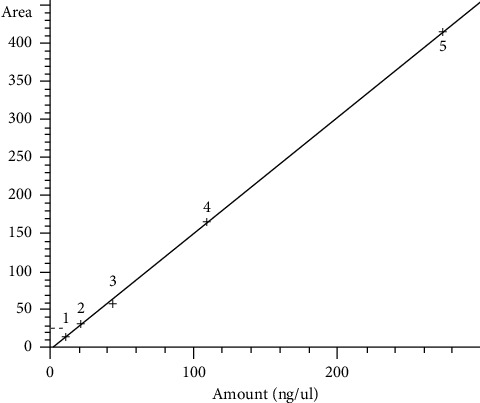
Calibration curve.

As can be seen in Figure 3, the calibration line had the nanograms injected on the x-axis and the area under the peak on the y-axis. The calibration curve and instrument sensitivity are acceptable when

1. The correlation of determination (*r*^2^) is greater than or equal to 0.99.
2. The peak relative to the 1^st^ point of the calibration curve is quantifiable.

**Figure 4:**
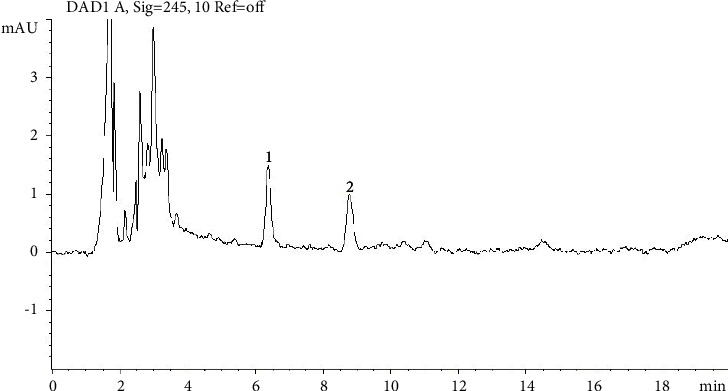
Chromatogram of a positive sample. Number 1 indicates doramectin peak (RT 6.372) and number 2 indicates ivermectin peak (RT 8.782).

The concentration of ivermectin present in the sample was calculated based on the photometric response obtained from the chromatogram report, as can be seen in Figure 4. In the case of positive samples above the maximum concentration, the sample was diluted to remain in the scope.

The concentration of ivermectin in the sample was calculated as shown in equation (1), where A is the injected amount of ivermectin (ng), *P* is the weight of the sample (g), and 2 is the result obtained from the simplification of the calculation: ng injected x 1000 *µ*I x 50 mL x 2 mL/100 *µ*L x P x 2 mL x 1000.

(1) $$\begin{array}{c} Concentration\left(\frac{mg}{kg}\right)=\frac{A}{\left(P*2\right)}\text{.} \end{array}$$

The concentration of ivermectin added to the fortified sample was calculated by interpolation on the calibration line.

To qualitatively confirm the presence of ivermectin, co-chromatography was carried out to make sure that the analyte in the suspect sample is indeed the one that is being investigated. The chromatography is run by injecting 50 *µ*L of a known sample extract together with a 50 *µ*L volume of one of the working solutions of ivermectin. The working solution is chosen so that it corresponds to the same amount in ng of analyte comparable to that present in the sample. The perfect overlap of the standard and sample confirms the association when the peak has a good shape, and it is symmetrical without splitting or any deformations that may indicate the presence of an interferent and not of an analyte. The area under the peak will be equal to the sum of half of the standard and half of the sample because 50 *µ*L of each was injected instead of a full injection of 100 *µ*L. The spectrum of the sample peak was also compared with the spectrum at intermediate concentration, evaluating the overlap factor (match factor), which must not be less than 900/1000.

### 3.1. Precision and Accuracy

The quantitative confirmatory method validation was performed according to the Annex III of the EU Regulation 2017/625. The measurement uncertainty was expressed, at the concentration subject to validation (detection limit of 0.5 mg/kg), using the *β* error (false negative) result less than or equal to 5% (statistical certainty 1 − *β* = 95%).

#### 3.1.1. Specificity, Detection, and Quantification

Specificity was assessed by applying the entire test method to 20 blank samples for both solid and liquid samples. None of the samples showed chromatographic peaks at the analyte retention time.

To verify detection capability, extracts of 20 blank sample and the relative 20 strengthened at a value of 0.5 mg/kg were treated and injected in 2 analytical sessions. In all the fortified samples, the chromatographic peak at the ivermectin retention time was highlighted, while the blank samples did not show any chromatographic peak at the ivermectin retention time. Therefore, the maximum tolerable error of 5% was respected. The limit of detection (LOD) was set at 0.25 mg/kg and tested experimentally.

The limit of quantification (LOQ) was established at 0.5 mg/kg. It was experimentally verified through tests on fortified feed at the limit of quantification in six independent replicas.

#### 3.1.2. Accuracy

Accuracy was assessed through the 3-level recovery rate within the scope of the method. Precision was assessed by calculating the standard deviation and the coefficient of variation (%) by conducting 6 replicate tests for each of the concentration levels.

Linearity was evaluated to understand the field of application of the method (0.5–10 mg/kg). During calibration, *r*^2^ obtained was 0.9999 for ivermectin. Specificity was assessed by applying the entire test method to 6 blank samples. The tests conducted made it possible to verify the absence of an instrumental response around the retention time.

#### 3.1.3. Uncertainty

Uncertainty was expressed as the maximum extended relative uncertainty. In expressing the uncertainty, the actual degrees of freedom (v), calculated using the Welch–Satterthwaite formula, the calculated coverage factor (k), and a 95% confidence level (*p*) were considered. These parameters are summarized in Table 1.

From the above uncertainty, we calculate the uncertainty of the result (C) using the following equation:

(2) $$\begin{array}{c} \overset{{}^{\circ}}{U}\left(\overset{®}{\text{y}}\;r\right)=C*\frac{\overset{{}^{\circ}}{U}\left(\overset{®}{\text{y}}\right)}{100}\text{.} \end{array}$$

### 3.2. Method Validation

The method was validated for both screening and confirmatory purposes.

For the screening validation, the selectivity, specificity, and quantification limit were verified by the 20 independent analyses of blank feed samples and 20 independent analyses of blank feed samples spiked at the quantification limit concentration settled at 0.5 mg/kg. The same was done for drinking water: 20 independent analyses of blank water samples and 20 independent analyses of blank water samples spiked at the quantification limit concentration settled at 0.5 mg/kg. The method was found to be applicable also to this matrix.

The applicability of the method was studied for feed matrixes, and the concentration range was settled from the limit of quantification, 0.5 mg/kg, up to 10 mg/kg, considering the content of ivermectin in medicated feed. Further tests demonstrated that this method is also useful when studying soil matrixes. Indeed, when the soil was dried and analyzed in the same way as feed, sufficient recovery rates were met in spiked sample.

Linearity was evaluated to cover the entire range of application of the method. For each calibration curve (Figure 3), five concentration levels were chosen (0.5, 1, 2, 5, and 12.5 mg/kg) and every level was replicated three times. The calibration curves were obtained using least squares regression analysis. The dataset obtained was investigated using the ANOVA approach. The elaboration showed that the linear regression was highly significant (*P* < 0.001), the intercept values were not significantly different from zero, and the correlation coefficient values (>0.999) indicated excellent linearity.

Selectivity and specificity were evaluated by analyzing six samples from different kinds of feed and by confirming the absence of analytical substances with retention times similar to ivermectin.

The detection limit was fixed and verified at half of the LOQ value by analyzing six spiked feed samples at 0.25 mg/kg.

The quantification limit was fixed and verified at 0.5 mg/kg at half of the minimum quantification level concentration recommended by CReAA. Other validated methods involving LC in different matrixes have reached lower sensitivity by using different detectors, for example, MS was used by Morbidelli et al. in plasma to reach a 0.5–20.0 ng/mL range [17], by Durden et al. who saw in dried blood spots concentration of 1 ng/mL [16], and by Duthal et al. who could detect a range of e 0.5–60 ppb in milk [20]. Åsbakk et al. and Iglesias et al. used a fluorescence detector reaching sensitivity at 5−2000 ng/g and 1–2000 ng/g, respectively [12, 13].

To determine trueness, precision, recovery, and repeatability, spiked blank feed samples were used because no certified reference material was available for ivermectin. In detail: blank feed samples were spiked, before the beginning of the extraction procedure, with ivermectin at three different concentration levels, and 6 independent replicas were carried out for each spiking level.

The results obtained are shown in Tables 2 and 3.

Ruggedness and reproducibility were evaluated using ongoing data. Ruggedness was verified by comparing processed quality control samples made by different operators under different environmental conditions and at different times with different batches of standards and laboratory materials. The analytical method was found to be rough, as the quality controls results are stable under the planned operating conditions. The reproducibility CV%, calculated from 40 ongoing quality control data, is 8.40%.

## 4. Conclusions

The HPLC method presented here allows the detection and quantification of ivermectin levels in different matrixes by creating a calibration curve and using doramectin as a process standard. This validated method is easy, cheap to use, precise, and repeatable, and it needs very small amounts of samples.

It could be easily used to detect ivermectin in solid and liquid matrixes for monitoring and environmental purposes. In fact, this method could be a reference method for ecotoxicity studies on the control of environmental pollutants based on ivermectin. Further studies could use the same analysis to extend the applicability to other macrocyclic lactones or similar molecules in the same matrixes.

## Figures and Tables

**Table 1:** Summary of uncertainty parameters.

Analyte	Validation range (mg/kg)	Extended relative uncertainty Ů (ӯ)	Degrees of freedom (v)	Coverage factor (k)
Ivermectin	0.5–10	12.56	24	2.06

**Table 2:** Data elaboration for each concentration level.

Concentration level (mg/kg)	Number of replicas	Recovery rate (%)	Relative standard deviation	Coefficient of variation (%)
0.5	6	91.8	2.99	3.26
2	6	98.3	1.67	1.70
10	6	96.1	3.10	3.20

**Table 3:** Total elaboration of 18 spiked feed samples.

Total recovery rate (%)	Total relative standard deviation	Total coefficient of variation (%)
95.4	3.77	3.95

## Data Availability

The data used to support the findings of this study are available from the corresponding author upon request.

## Abbreviations

CV: Coefficient of variation
DAD: Diode array detector
ESI: Electrospray ionization
HPLC: High-performance liquid chromatography
LC: Liquid chromatography
LOD: Limit of detection
LOQ: Limit of quantification
MRLs: Maximum residual limits
MS: Mass spectrometry
RT: Retention time
SPE: Solid phase extraction.
